# Inter-rater reliability of subthreshold psychotic symptoms in individuals with 22q11.2 deletion syndrome

**DOI:** 10.1186/s11689-021-09372-3

**Published:** 2021-06-14

**Authors:** Tyler M. Moore, Deby Salzer, Carrie E. Bearden, Monica E. Calkins, Wendy R. Kates, Leila Kushan, Robert Sean Gallagher, Dafna Sofrin Frumer, Ronnie Weinberger, Donna M. McDonald-McGinn, Raquel E. Gur, Doron Gothelf

**Affiliations:** 1 Department of Psychiatry, Perelman School of Medicine, University of Pennsylvania, Philadelphia, Pennsylvania USA; 2 Lifespan Brain Institute of Penn Medicine and the Children’s Hospital of Philadelphia (CHOP), Philadelphia, USA; 3 The Behavioral Neurogenetics Center, Edmond and Lily Safra Children’s Hospital, Sheba Medical Center, Tel Hashomer, Israel; 4 Semel Institute for Neuroscience and Human Behavior, University of California, Los Angeles, USA; 5 Department of Psychiatry and Behavioral Sciences, State University of New York at Upstate Medical University, Syracuse, NY USA; 6 22q and You Center, CHOP, Philadelphia, USA; 7 Department of Child and Adolescent Psychiatry and Behavioral Sciences, CHOP, Philadelphia, USA; 8 Sackler Faculty of Medicine and the Sagol School of Neuroscience, Tel Aviv University, Tel Aviv, Israel

**Keywords:** Velocardiofacial syndrome, DiGeorge, Subthreshold psychotic symptoms, Structured Interview for Prodromal Syndromes (SIPS), Scale of Prodromal Symptoms (SOPS), Inter-rater reliability, Psychosis risk syndrome

## Abstract

**Background:**

Pathways leading to psychosis in 22q11.2 deletion syndrome (22q11.2DS) have been the focus of intensive research during the last two decades. One of the common clinical risk factors for the evolution of psychosis in 22q11.2DS is the presence of positive and negative subthreshold psychotic symptoms. The gold standard for measuring subthreshold symptoms is the Structured Interview for Prodromal Syndromes (SIPS) and its accompanying Scale of Prodromal Symptoms (SOPS) ratings. Although the scale has been used by many centers studying 22q11.2DS, the inter-site reliability of the scale in this population has never been established.

**Methods:**

In the present study, experienced clinical assessors from three large international centers studying 22q11.2DS independently rated video recordings of 18 adolescents and young adults with 22q11.2DS.

**Results:**

The intraclass correlations coefficients (ICCs) among three raters for the SOPS total scores, as well as for the positive, negative, and disorganization subscale scores, were good-to-excellent (ICCs range 0.73–0.93). The raters were also able to reliably determine the subjects’ subthreshold syndrome status (ICC = 0.71). The reliability of individual items was good-to-excellent for all items, ranging from 0.61 for motor disturbances [G3] to 0.95 for bizarre thinking.

**Conclusions:**

Our results show that trained clinicians can reliably screen for subthreshold psychotic symptoms in individuals with 22q11.2DS. To increase assessment reliability, we suggest specific clarifications and simplifications to the standard SIPS interview for future studies.

**Supplementary Information:**

The online version contains supplementary material available at 10.1186/s11689-021-09372-3.

## Background

The 22q11.2 deletion syndrome (22q11.2DS), also known as DiGeorge or velocardiofacial (VCFS) syndrome, is among the most common microdeletion genetic disorder characterized by a variety of medical, cognitive, and psychiatric manifestations [1, 2]. One of the most frequent co-occurring psychiatric disorders in 22q11.2DS is schizophrenia-like psychotic disorder [3, 4]. In comparison to a lifetime prevalence of ~ 3% in the general population [5], up to 40% of persons with 22q11.2DS develop psychotic disorders [4].

Schizophrenia is a neurodevelopmental disorder with a prodromal phase of milder symptoms, including disturbances in perception thought processes, with yet intact reality testing. These symptoms are considered “attenuated positive symptoms.” The Structured Interview for Prodromal Symptoms (SIPS) was developed to objectively quantify the attenuated psychotic symptoms that are subthreshold and present in the clinical high risk (CHR) state [6]. The conversion rate from CHR to schizophrenia is about 15–20% per year [7]. Because psychotic disorders are prevalent in 22q11.2DS, research efforts are devoted to identifying early signs and predictors of psychosis in this population, leading to the study of attenuated psychotic symptoms in 22q11.2DS [8–17].

Weisman et al. [17] analyzed data on subthreshold psychosis in 760 individuals, ages 6 to 55 years, with 22q11.2DS from 10 centers. The highest rate of subthreshold psychotic symptoms was found in adolescence and young adulthood, with about one-third of adolescents and young adults with 22q11.2DS meeting criteria for positive and negative/disorganized subthreshold symptoms. The annual conversion rate to psychotic disorder for individuals with 22q11.2DS and positive subthreshold symptoms, as reported in longitudinal studies, is between 9% and 15% [14, 18, 19]. Accumulating evidence suggests that negative subthreshold symptoms in 22q11.2DS are clinically significant, as they are associated with the severity of neurocognitive deficits [17, 19] and with the presence of anxiety disorders and attention deficit/hyperactivity disorder (ADHD) [19]. Furthermore, the severity of some negative symptoms has been shown to be a significant predictor of transition to psychosis [20].

The above-mentioned studies used the Structured Interview for Prodromal Syndromes (SIPS) and the Scale of Prodromal Symptoms (SOPS) to measure subthreshold symptoms. Semi-structured diagnostic interviews like the SIPS are the gold standard instruments for evaluating attenuated psychotic symptoms in the general population [21, 22]. Several studies have supported its inter-rater reliability and predictive validity in typical help-seeking persons [23–26]. Yet, to date, no study has established the inter-rater reliability of this commonly used assessment instrument in 22q11.2DS. Lack of reliability in using the SIPS in this population might partially explain the high variability in the rates of subthreshold symptoms of psychosis, as determined by the SIPS/SOPS, among 22q11.2DS cohorts. The rates of positive subthreshold psychotic symptoms varied among studies from 20 to 56% (see [15] Table 1). Of note are the complexities of assessing subthreshold psychotic symptoms in individuals with neurodevelopmental disorders such as 22q11.2DS, which often co-occur with cognitive disabilities and medical issues. Taken together, it seems essential to verify the inter-rater reliability of the SIPS in 22q11.2DS.

**Table 1:** Demographic and clinical characteristics of the study sample

Characteristic	Value
Number^a^ of subjects (% females)	18 (72)
Mean age ± SD (range)	21.72 ± 7.47 (12–36)
Mean years of education ± SD (range)	11.71 ± 3.85 (5–18)
Mean FSIQ ± SD (range)	83.20 ± 13.44 (66–110)
Mean GAF ± SD (range)	57.89 ± 17.15 (33–87)
Number of subjects with a psychiatric diagnosis (%)	16 (89)
Any anxiety disorder	10
Any mood disorder	4
ADHD	9
Learning disability	2
ASD	2
Number of subjects with more than one psychiatric diagnosis	10
Number of subjects with a psychiatric medication (%)	14 (78)
Benzodiazepine	3
SSRI	7
Other antidepressants	3
Stimulant	4
Alpha-2 agonist	3
SNRI	2
Anticonvulsant	2
Atypical antipsychotic	2
Number of subjects with more than one class of psychiatric medication	8

^a^One subject was from the Tel-Aviv site, six were from Penn and the rest were assessed at UCLA; *FSIQ* Full-Scale IQ, *GAF* Global Assessment of Functioning, *ADHD* attention deficit/hyperactivity disorder, *ASD* autism spectrum disorder, *SSRI* selective serotonin reuptake inhibitor, *SNRI* selective norepinephrine reuptake inhibitor

The overarching aim of the present study was to establish cross-site reliability for the SIPS interview and SOPS scoring in individuals with 22q11.2DS. For this purpose, clinical assessors from three international centers with experience both with the SIPS interview and with 22q11.2DS independently rated video recordings of adolescents and young adults with 22q11.2DS. Intraclass correlation coefficients (ICCs) among three raters were assessed.

## Method

### Participants

The demographic and clinical characteristics of the study sample are outlined in Table 1. Videotaped SIPS interviews of 18 individuals with 22q11.2DS were conducted among 22q11.2DS patients recruited from three established international centers—Children’s Hospital of Philadelphia (CHOP) and University of Pennsylvania (Penn), University of California Los Angeles (UCLA), and Sheba Medical Center in Tel-Aviv. The individuals selected for recording were consecutive presentations to the participating sites with good recording quality. They are representative of youth with the syndrome who can assent/consent and engage in research. All study participants were Caucasian. Inclusion criteria included genetically confirmed diagnosis of 22q11.2DS by fluorescent in situ hybridization, chromosomal microarray, and/or multiplex ligation probe amplification [27]; verbal IQ > 70; and willingness of participants (and their parents in cases of minors) to have the interview video-recorded and shared with collaborators for scoring. All participants (and their parents, if involving minors) received a full explanation about the study and provided their written informed assent/consent to participate in the study. This study was approved by the Institutional Review Boards of Sheba Medical Center, Penn, and UCLA.

### Cognitive and psychiatric assessments

Cognitive assessment was performed using the Wechsler Intelligence Scale for Children, 3rd edition (WISC III) [28] for participants aged ≤ 17 years or the Wechsler Adult Intelligence Scale, 3rd edition (WAIS III) [29] for participants aged > 17.

Psychiatric evaluation for children and adolescents was carried out using a modified version of the Kiddie Schedule for Affective Disorders and Schizophrenia for School Age Children-Present and Lifetime Version (K-SAD-PL) [30]. Adults (age > 18) were also evaluated by the Structured Clinical Interview for DSM-IV Diagnoses (SCID) as previously described [17].

### Assessment of subthreshold psychotic symptoms

Assessment of subthreshold psychotic symptoms was conducted using the SIPS interview and its accompanied scoring—the SOPS [31]. The SOPS is composed of 19 items, each representing 1 out of 4 domains—subscales of attenuated psychotic symptoms: positive (5 items), negative (6 items), disorganization (4 items), and general (4 items) symptoms subscales [31]). The scale specifies severity scoring accompanied by anchoring criteria for each item, ranging from 0 (absent) to 6 (severe and psychotic/extreme.

Eighteen consecutive 22q11.2DS individuals agreeing to be video-recorded were enrolled to the study. Participants were interviewed by skilled masters level and experienced interviewers who underwent training and achieved certification at their respective institution in SIPS administration as part of ongoing collaborative studies. All interviews were in English except for one patient who was recruited from Israel. That interview was translated to English (by REG) to enable the rating by the US clinicians. The interviews’ video recordings were uploaded to a secure website and were downloaded and scored, and these scorings were used for calculating the inter-rater reliabilities. The video recordings were accompanied by a short clinical summary, which included brief participant background information—age, education, brief medical history, living situation, and cognitive functioning. Each recording was independently rated by three senior doctoral level clinicians, one from each center, who were not familiar with the SIPS ratings of the participants. These raters are experienced in standardized psychiatric assessment of individuals with 22q11.2DS—a child psychiatrist (DG) and two clinical psychologists (CEB and MEC). SOPS scorings of each interview were entered in the REDCap database independently by each faculty rater. Importantly, the rater was not familiar with the rated participants prior to rating the interview. Monthly telephone meetings were held with the participation of the three assessors and REG. At all telephone meetings the independent scorings of each item were discussed. Items without consensus in scoring were discussed to reach a consensus, which was determined to serve as the gold standard scoring in future usage of the videos for training purposes.

Based on each rater’s independent scores of the individual SOPS items, and in line with earlier work [25], summary measures were derived for further analyses: (1) total SOPS score was calculated by summing the 19 items ratings; (2) total score for each SOPS subscale was calculated by summing relevant item scores per subscale (positive, negative, disorganization, and general).

The presence of subthreshold psychotic symptoms was determined by summing up item ratings of positive, negative, and disorganization subscales. Four categories of subthreshold symptoms were calculated as previously described [17, 32]: (1) *positive subthreshold symptoms*—at least one positive symptom rated 3–5 (3, moderate; 4, moderately severe; 5, severe but not psychotic); (2) *negative/disorganized subthreshold symptoms*—at least two or more negative/disorganized symptoms rated 3–6, in the absence of positive symptoms; (3) *positive + negative/disorganized subthreshold symptoms*—at least one positive symptom rated 3–5 and at least two negative/disorganized symptoms rated 3–6; and (4) *Acute positive subthreshold symptoms—*at least one positive symptom with a rating of 6.

### Statistical analyses

All analyses were conducted using SPSS and the *irrNA* package [33] in R [34]. SOPS’ endpoint measures were analyzed for inter-rater reliability by Intra class Correlations Coefficients (ICCs) [two-way random model; single measurements form; absolute agreement type]. ICCs were estimated using the Brueckl [35] method, which handles missing data without imputation or data loss. Reliability levels were interpreted according to the following ICC guidelines [35, 36] representing different levels of agreement among judges: < 0.4—low; 0.40–0.59—fair; 0.60–0.74—good; and ≥ 0.75—excellent. ICCs were calculated on a max of 14 subjects (100% valid cases). Finally, to explore the extent to which our results are influenced by the relatively low symptom levels in this sample, the above analyses were repeated after splitting the sample into clinical high risk (CHR; many symptoms) and non-CHR (few or no symptoms).

## Results

### Distribution of subthreshold psychotic symptoms based on consensus scores

SOPS’ gold standard ratings for the 18 interviews were used to analyze the prevalence rates of subthreshold psychotic symptoms in the current sample. Thirty-nine percent of the sample (*n* = 7) had no subthreshold psychotic symptoms, 33% had negative/disorganized symptoms (*n* = 6), 17% (*n* = 3) positive and positive + negative/disorganized subthreshold symptoms were present in *two subjects (11%).*

### Inter-site reliability of the SOPS and its subscales

ICCs of the inter-rater reliability analyses conducted on the total scores of the SOPS and its four subscales are shown in Table 2. Results revealed excellent agreement among raters with respect to the overall SOPS and all subscales [ICCs: 0.73–0.96].

**Table 2:** Interclass correlation coefficients (ICCs) of the total SOPS scale and subscales

Item	Description	ICC (95 % CI)	% valid cases
Total SOPS	Scale of prodromal syndromes	0.957 (0.906–0.983)	100
Total D	Disorganization symptoms subscale	0.923 (0.840–0.968)	100
Total P	Positive symptoms subscale	0.894 (0.784–0.955)	100
Total N	Negative symptoms subscale	0.889 (0.778–0.953)	100
Total G	General symptoms subscale	0.729 (0.513–0.877)	100

*CI* = confidence interval, 100% valid cases refer to *n* = 18

### Inter-site reliability of the SOPS’ individual items

The inter-rater agreement for each item of the SOPS is shown in Table 3. For 17 out of the 19 SOPS items, there was excellent agreement among raters [ICCs 0.76–0.95]. Two items had good agreement [ICCs: 0.61–0.65]. For finer-grained comparisons, Supplementary Table S2 shows the mean, minimum, and maximum rating of each rater for each item. Note that several items had less than 100% valid data. The interviews were conducted with complex patients that commonly manifest short attention span and restlessness and therefore for a few patients the last questions of the SIPS interview had to be shortened. In these cases, the raters decided that the information in the video is insufficient to provide a valid score and these items were left as “missing.” Furthermore, it is important to note that for the “positive,” “negative”, and “disorganized” items the percent of valid cases was between 93 and 100%. The lower rates were for the “general” items, which are of minor importance in the SIPS interview as they are not part of the definition of clinical high risk state.

**Table 3:** Intraclass correlation coefficients (ICCs) of the SOPS’ 19 individual items

Item	Description	ICC (95 % CI)	% valid cases
D2	Bizarre thinking	0.945 (0.883–0.977)	96
N4	Experience of emotions and self	0.938 (0.868–0.974)	94
P4	Perceptual abnormalities/hallucinations	0.923 (0.842–0.968)	100
G4	Impaired tolerance to normal stress	0.912 (0.811–0.964)	89
P2	Suspiciousness/persecutory ideas	0.899 (0.795–0.958)	98
N6	Occupational functioning	0.886 (0.759–0.953)	93
D3	Trouble with focus and attention	0.864 (0.706–0.944)	96
P1	Unusual thought content/delusional ideas	0.852 (0.707–0.937)	98
N5	Ideational richness	0.819 (0.654–0.921)	98
G2	Dysphoric mood	0.812 (0.582–0.925)	85
N1	Social anhedonia	0.811 (0.635–0.918)	98
N3	Expression of emotion	0.809 (0.630–0.917)	96
G1	Sleep disturbance	0.769 (0.562–0.898)	96
D1	Odd behavior or appearance	0.765 (0.567–0.895)	100
N2	Avolition	0.764 (0.550–0.897)	94
D4	Personal hygiene	0.763 (0.555–0.896)	94
P5	Disorganized communication	0.758 (0.551–0.892)	100
P3	Grandiosity	0.645 (0.392–0.833)	96
G3	Motor disturbances	0.606 (0.325–0.814)	94

*CI* confidence interval, 100% valid cases refer to *n* = 18

### Inter-site reliability of the SOPS in assessing subthreshold symptoms status

**Fig. 1:**
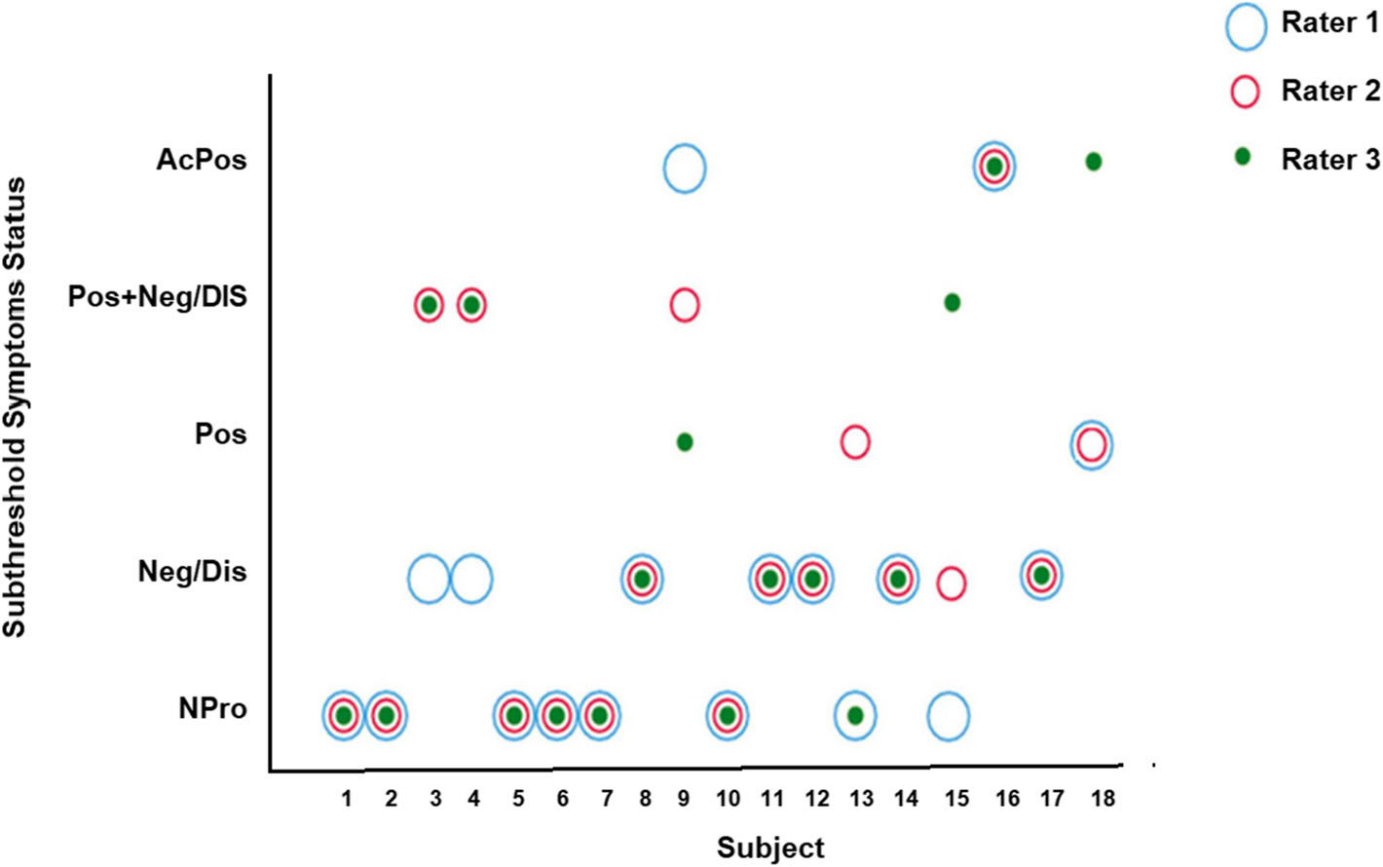
Inter-rater agreement between three sites regarding the subthreshold symptoms status of each subject. Npro: non-prodromal; Neg/Dis: negative/disorganized subthreshold symptoms; Pos: positive subthreshold symptoms; Pos + Neg/Dis: positive and negative/disorganized subthreshold symptoms; AcPos: acute positive subthreshold symptoms. Note that a 1-point difference in one item can change the status of the patient from one category to the other. In case of subject#9, there was a 1-point difference in the score of one positive symptom of the SIPS—one rater gave a score of 6 “acute positive” and the other two gave a score of ≤ 5 within the positive (subthreshold) range; and 1-point difference in the negative/disorganized symptoms—one rater gave a score of ≥ 3 for only one negative/disorganized symptom and two raters had a score of ≥ 3 only for one negative/disorganized symptom

Finally, to further test whether the raters were able to distinguish reliably between subthreshold symptoms and non-clinically significant symptoms, ICC was computed on the combined total scores of three SOPS subscales: positive, negative, and disorganized symptoms. As seen in Fig. 1, agreement among raters was found to be in the good range for determining negative/disorganized subthreshold symptoms, positive subthreshold symptoms, positive and negative/disorganized subthreshold symptoms, acute positive subthreshold symptoms, or as not psychosis-prone ICC = 0.71 [95% CI 0.47–0.89].

### Comparison of inter-site reliability in CHR and non-CHR

Supplementary Table S1 shows the same results as in Table 3 but split by CHR status (11 CHR compared to 7 non-CHR). Of the 14 items comparable across samples, six showed higher ICCs in the non-CHR sample. Item N3 showed the largest difference (0.33 in non-CHR, compared to 0.93 in CHR), while item G2 showed the largest difference in the opposite direction (0.92 in non-CHR, compared to 0.75 in CHR).

## Discussion

To our knowledge, this is the first study to test SIPS/SOPS inter-rater reliability in 22q11.2DS. The few SIPS reliability studies have been conducted only by the developers of the tool and only in US clinical high risk, help-seeking individuals, without a neurogenetic syndrome [25, 26]. Thus, our study is also the first inter-rater study of individuals with developmental disabilities. To date, there is no reliability study assessing subthreshold psychotic symptoms not only for 22q11.2DS but also for any neurogenetic syndrome. In this study, international experts from three large academic centers studying 22q11.2DS were able to obtain overall good inter-rater reliability. These efforts can advance the development of guidelines and gold standard assessment training videos, which can be useful for other researchers using the SIPS in evaluating 22q11.2DS individuals. Our results and the accompanying guidelines are relevant also for researchers studying individuals with other genetic syndromes associated with increased risk for psychosis such as Prader-Willi syndrome and 16p11.2 duplication syndrome [36]. Establishing reliability for measuring subthreshold symptoms can benefit treatment studies since the tool can be used to obtain outcome measures to determine efficacy of intervention. Importantly, information on reliability will help contextualize studies in neurogenetic populations with those carried out in community samples.

We found excellent agreement among raters (ICCs > 0.75) for total SOPS’ scores and for all subscales and good reliability (ICC = 0.71, Fig. 1) in distinguishing between the various subthreshold syndromes. These results are in line with those demonstrated by Miller et al. [25] in clinical high-risk, help-seeking non-22q11.2DS population. While our sample was relatively small it was almost 30% larger (*n* = 18) than that of Miller et al. [25] (*n* = 14). Inter-rater reliability was at least in the “good” range for all SOPS items, where 17 of those 19 were in the “excellent” range. Of note, ICC values are inherently sensitive to the level of variability among participants’ scores (i.e., higher between-subjects variability improves inter-rater reliability coefficients) [37].

A limitation of our study was that we did not employ the traditional two-step process used in some reliability studies. At the first step, similarly to what we did in our study, after raters score interviews independently, they discuss differences in ratings to improve consensus and reach a set of agreed criteria. In the second step, not done in our study, these criteria are used by a new, other group of raters that score interviews without any discussion, thereby avoiding self-biases. Thus, additional study is recommended with a new sample of 22q11.2DS assessments and other raters.

Our data were acquired from three sites, but 11 out of the 18 subjects were from one site (UCLA). Each research group likely has some site-specific practices in its method of interviewing individuals with 22q11.DS, which can in turn influence the scoring. Thus, the over-representation of subjects from one site might somewhat bias our results. Future studies should expand the number of participants and include participants that are well-distributed across a variety of sites.

Notably, in this study IQ > 70 was an inclusion criterion and the conclusions may not extend to individuals with cognitive disability. Based on our experience, we have suggestions for administering the SIPS and assessing symptoms in individuals with 22q11.2DS, consisting of (1) clarification/simplification of interview questions, (2) elaboration of qualifiers with examples, and (3) use of numerical scales to assist the participant in rating his/her symptoms.

For example, in assessing grandiose ideas (P3), the interview includes the questions “Have you ever behaved without regard to painful consequences? For example, do you ever go on excessive spending sprees?” We recommend stating the question as it is written, but following the question up with clarifications / simplifications of language consisting of “In other words, have you ever behaved without thinking about the bad things that could happen because of what you are doing? For example, have you ever spent a lot of money on things you can’t afford?”

In assessing suspiciousness/persecutory ideas, the interview begins with the question: “Do you ever feel that people around you are thinking about you in a negative way” and follows the question up with qualifiers that assess onset-duration, degree of distress, degree of interference with life, and degree of conviction/meaning. For individuals with cognitive disabilities, we recommend that the interviewer elaborate on and concretize these qualifiers and provide numerical scales for some of the probes. For example, in probing for when symptoms began, we suggest providing a specific timepoint or life event anchor that would be relevant to the participant (e.g., “Did this start before or after you began middle school?”). In probing for degree of distress, we suggest that the interviewer follow up the interview question “What is this experience like for you? Does it bother you” with “On a scale from 0 to 10, with 10 being the most upsetting; how upsetting would you rate this?” Visual representations of such a scale can also be used, which permit the participant to point to the rating with which s/he agrees. We have modified the SIPS to reflect these and other guidelines in an effort to optimize its administration for individuals with cognitive disability. (Our full protocol of suggested modifications for individuals with cognitive disability can be obtained from the authors).

## Conclusions

To summarize, given the high rates of subthreshold psychotic symptoms in 22q11.2DS individuals, it is of utmost importance to validate the SIPS in the 22q11.2DS population. Our preliminary results with three international assessors reviewing SIPS video assessments of 18 22q11.2DS individuals suggest that good inter-reliability can be achieved for most SOPS items.

## Supplementary Information

**Additional file1: Table S1.** Intraclass Correlation Coefficients (ICCs) of the SOPS’ 19 individual items. **Table S2.** Mean Itemwise and Overall Mean SOPS Ratings, by Rater.

## Data Availability

The datasets used and/or analyzed for the present paper can be made available upon a reasonable request to the corresponding author.

## Abbreviations

22q11.2DS: 22q11.2 deletion syndrome
ADHD: Attention deficit/hyperactivity disorder
ASD: Autism spectrum disorder
CHOP: Children’s Hospital of Philadelphia
FSIQ: Full-Scale IQ
GAF: Global Assessment of Functioning
ICCs: Intraclass correlations coefficients
K-SAD-PL: The Kiddie Schedule for Affective Disorders and Schizophrenia for School Age Children-Present and Lifetime Version
SCID: The Structured Clinical Interview for DSM-IV Diagnoses
SIPS: Structured Interview for Prodromal Syndromes
SNRI: Selective norepinephrine reuptake inhibitor
SOPS: Scale of Prodromal Symptoms
SSRI: Selective serotonin reuptake inhibitor
UCLA: University of California Los Angeles
VCFS: Velocardiofacial syndrome
WAIS III: Wechsler Adult Intelligence Scale, 3rd edition
WISC III: Wechsler Intelligence Scale for Children, 3rd edition
