# Supplementary material for: Inter-rater reliability of subthreshold psychotic symptoms in individuals with 22q11.2 deletion syndrome
Source: J Neurodev Disord. 2021 Jun 14;13:23. doi: 10.1186/s11689-021-09372-3 (PMC8204529; doi:10.1186/s11689-021-09372-3)
Supplement: Supplementary file 1 — Additional file1: Table S1. Intraclass Correlation Coefficients (ICCs) of the SOPS’ 19 individual items. Table S2. Mean Itemwise and Overall Mean SOPS Ratings, by Rater. [file 11689_2021_9372_MOESM1_ESM.docx]

| **Table S1** Intraclass Correlation Coefficients (ICCs) of the SOPS' 19 individual items | | |
| --- | --- | --- |
|  | ICC (95 % CI) | |
| Item | Non-CHR | CHR |
| D1 | n/a | 0.769 (0.505 - 0.924) |
| D2 | n/a | 0.937 (0.837 - 0.981) |
| D3 | 0.917 (0.731 - 0.984) | 0.815 (0.554 - 0.942) |
| D4 | n/a | 0.644 (0.298 - 0.876) |
| G1 | 0.903 (0.682 - 0.981) | 0.717 (0.398 - 0.905) |
| G2 | 0.924 (0.732 - 0.985) | 0.747 (0.413 - 0.923) |
| G3 | 0.546 (0.015 - 0.892) | 0.651 (0.313 - 0.878) |
| G4 | 0.811 (0.452 - 0.961) | 0.918 (0.787 - 0.975) |
| N1 | 0.453 (0.011 - 0.851) | 0.840 (0.630 - 0.949) |
| N2 | 0.688 (0.290 - 0.929) | 0.682 (0.322 - 0.893) |
| N3 | 0.333 (-0.084 - 0.799) | 0.925 (0.808 - 0.977) |
| N4 | n/a | 0.940 (0.843 - 0.982) |
| N5 | 0.649 (0.181 - 0.920) | 0.807 (0.566 - 0.938) |
| N6 | 0.609 (0.185 - 0.905) | 0.874 (0.649 - 0.963) |
| P1 | 0.868 (0.614 - 0.973) | 0.825 (0.588 - 0.945) |
| P2 | 0.789 (0.457 - 0.955) | 0.895 (0.742 - 0.968) |
| P3 | n/a | 0.663 (0.330 - 0.883) |
| P4 | 0.899 (0.692 - 0.980) | 0.912 (0.781 - 0.973) |
| P5 | 0.800 (0.457 - 0.958) | 0.727 (0.426 - 0.908) |
| Total D | 0.933 (0.781 - 0.987) | 0.878 (0.708 - 0.962) |
| Total G | 0.643 (0.211 - 0.917) | 0.779 (0.478 - 0.930) |
| Total N | 0.450 (0.004 - 0.851) | 0.865 (0.685 - 0.958) |
| Total P | 0.903 (0.692 - 0.981) | 0.868 (0.682 - 0.959) |
| Total SOPS | 0.826 (0.483 - 0.965) | 0.941 (0.852 - 0.982) |
| Note. CI = Confidence Interval; CHR = clinical high-risk; n/a values are due to insufficient variability in rater responses (e.g. all raters rate "0"), and thus n/a values could be thought of as perfect agreement. | | |

| **Supplementary Table S2** Mean itemwise and overall mean SOPS ratings, by rater | | | | |
| --- | --- | --- | --- | --- |
| Rater & Item | n | mean | min | max |
| Rater 1: p1 | 18 | 1.17 | 0 | 5 |
| Rater 2: p1 | 17 | 1.18 | 0 | 5 |
| Rater 3: p1 | 18 | 1.22 | 0 | 6 |
| Rater 1: p2 | 18 | 0.94 | 0 | 5 |
| Rater 2: p2 | 17 | 1.06 | 0 | 5 |
| Rater 3: p2 | 18 | 0.89 | 0 | 6 |
| Rater 1: p3 | 18 | 0.39 | 0 | 2 |
| Rater 2: p3 | 16 | 0.19 | 0 | 2 |
| Rater 3: p3 | 18 | 0.17 | 0 | 1 |
| Rater 1: p4 | 18 | 1.44 | 0 | 6 |
| Rater 2: p4 | 18 | 1.50 | 0 | 6 |
| Rater 3: p4 | 18 | 1.61 | 0 | 6 |
| Rater 1: p5 | 18 | 1.11 | 0 | 5 |
| Rater 2: p5 | 18 | 0.78 | 0 | 4 |
| Rater 3: p5 | 18 | 1.33 | 0 | 4 |
| Rater 1: n1 | 18 | 1.28 | 0 | 4 |
| Rater 2: n1 | 17 | 1.24 | 0 | 5 |
| Rater 3: n1 | 18 | 1.22 | 0 | 4 |
| Rater 1: n2 | 18 | 1.61 | 0 | 4 |
| Rater 2: n2 | 17 | 1.00 | 0 | 3 |
| Rater 3: n2 | 16 | 1.38 | 0 | 4 |
| Rater 1: n3 | 17 | 1.06 | 0 | 4 |
| Rater 2: n3 | 17 | 1.12 | 0 | 4 |
| Rater 3: n3 | 18 | 1.17 | 0 | 4 |
| Rater 1: n4 | 18 | 0.56 | 0 | 4 |
| Rater 2: n4 | 17 | 0.65 | 0 | 4 |
| Rater 3: n4 | 16 | 0.56 | 0 | 5 |
| Rater 1: n5 | 18 | 2.06 | 0 | 4 |
| Rater 2: n5 | 17 | 1.65 | 0 | 4 |
| Rater 3: n5 | 18 | 1.94 | 0 | 5 |
| Rater 1: n6 | 18 | 1.39 | 0 | 6 |
| Rater 2: n6 | 15 | 1.33 | 0 | 6 |
| Rater 3: n6 | 17 | 0.88 | 0 | 5 |
| Rater 1: d1 | 18 | 0.33 | 0 | 2 |
| Rater 2: d1 | 18 | 0.33 | 0 | 3 |
| Rater 3: d1 | 18 | 0.44 | 0 | 4 |
| Rater 1: d2 | 18 | 0.39 | 0 | 4 |
| Rater 2: d2 | 18 | 0.33 | 0 | 4 |
| Rater 3: d2 | 16 | 0.44 | 0 | 4 |
| Rater 1: d3 | 18 | 1.89 | 0 | 5 |
| Rater 2: d3 | 17 | 1.59 | 0 | 5 |
| Rater 3: d3 | 17 | 2.24 | 0 | 5 |
| Rater 1: d4 | 18 | 1.11 | 0 | 4 |
| Rater 2: d4 | 16 | 1.06 | 0 | 4 |
| Rater 3: d4 | 17 | 0.94 | 0 | 3 |
| Rater 1: g1 | 18 | 1.78 | 0 | 3 |
| Rater 2: g1 | 16 | 1.38 | 0 | 3 |
| Rater 3: g1 | 18 | 1.28 | 0 | 4 |
| Rater 1: g2 | 17 | 1.41 | 0 | 5 |
| Rater 2: g2 | 15 | 1.07 | 0 | 4 |
| Rater 3: g2 | 14 | 0.64 | 0 | 3 |
| Rater 1: g3 | 17 | 0.53 | 0 | 2 |
| Rater 2: g3 | 17 | 0.47 | 0 | 3 |
| Rater 3: g3 | 17 | 0.47 | 0 | 2 |
| Rater 1: g4 | 18 | 1.22 | 0 | 6 |
| Rater 2: g4 | 15 | 1.27 | 0 | 5 |
| Rater 3: g4 | 15 | 1.07 | 0 | 5 |
| Rater 1: Mean Overall | 18 | 1.14 | 0.16 | 3.63 |
| Rater 2: Mean Overall | 18 | 1.01 | 0.07 | 3.11 |
| Rater 3: Mean Overall | 18 | 1.06 | 0.21 | 3.28 |
